# Dr. Wm. P. Greenwood and Mr. Peabody

**Published:** 1842-09

**Authors:** 


*Dr. Wm. P. Greenwood and Mr. Peabody* were both present and participated in the proceedings of the American Society of Dental Surgeons, at their late meeting in Boston. It was gratifying to meet with these venerable practitioners. They seemed to take as lively an interest in the doings of the association as did any of the younger members. Dr. Greenwood has been a practitioner for upwards of fifty years, and although he is eighty years of age, his step is still elastic, and his mind possessed of the vigour and freshness of youth. After the adjournment of the evening session of the first day of the meeting, he gave a highly interesting account of the state of the profession fifty years ago, in this country. At that time, he said, the meaning of the word dentist, in many places, was hardly known, and consequently, little or no attention was paid to the use of the means for the preservation of the teeth, to the cure of their diseases, or to the supplying of their places with artificial ones when lost.

Dr. Greenwood presented to the society a specimen of mineral artificial teeth, manufactured and inserted by M. Debois de Chemant, of Paris, a resident in London in the year 1797. They were made for a lady of Boston, at that time in London, and worn by her for three years, when they were accidentally broken. On her return to Boston in 1800, Dr. Greenwood made a set from osseous substance to supply their place, and she left the mineral ones with him. They were the first of the kind which he had ever seen. He also presented to the society one of De Chemant's Dissertations on Artificial Teeth.

We hope that Drs. Greenwood and Peabody may live to attend many meetings of the society, and until it shall have accomplished the object for which it was formed.

